# Supplementary material for: A Triple Gene-Deleted Pseudorabies Virus-Vectored Subunit PCV2b and CSFV Vaccine Protect Pigs against a Virulent CSFV Challenge
Source: Viruses. 2023 Oct 25;15(11):2143. doi: 10.3390/v15112143 (PMC10674279; doi:10.3390/v15112143)
Supplement: Supplementary file 1 [file viruses-15-02143-s001.zip › viruses-2629202-supplementary.pdf]

Supplementary table S1: Classical swine fever virus (CSFV)-specific serum neutralizing (SN) antibody titer developed in pigs after PRVtmv+ vaccination

Group	#	CSFV-Neutralizing antibody titer (days post immunization)	
		Day 0	Day 28
PRVtmv+	**84**	<1	32
	**87**	<1	16
	**88**	<1	32
	**91**	<1	32
	**92**	<1	16
	**Average**	<1	25.6

Supplementary table S2: Clinical evaluation: Body temperature of pigs following the classical swine fever virus (CSFV) challenge.

Days post challenge	Pigs Temperature in °C									
	CSFV control					PRVtmv+				
	83	85	86	89	90	84	87	88	91	92
0	38.72	38.94	39.83	39.72	39.88	39.27	40.16	39.55	38.77	39.38
1	39.11	39.27	39.44	39.55	39.33	39	39.5	39.44	38.55	39
2	39	39.44	39.33	39.44	39.11	39.33	39.33	39.27	38.88	39.27
3	39.33	39.11	39.5	39.55	39.33	39.5	39.27	39.44	39.27	39.5
4	39.5	39.27	39.33	39.44	39.27	39.11	39.55	39.11	39.22	39.88
5	39.5	40.61	40.94	40.88	41.11	39.38	39.77	39.5	39.5	40.88
6	40.27	41	41.11	40.61	40.5	39.22	39.77	39.55	39.16	39.77
7	39.5	-	40.94	40.88	41.11	39.5	39.27	39.11	39.55	39.72
8						40.22	39.83	39.66	39.38	39.5
9						38.61	38.77	38.66	38.55	38.55
10						38.94	39.38	39.27	38.72	38.94
11						39.22	39.11	38.5	38.94	38.44
12						38.77	38.88	38.44	39.11	38.72
13						39	39.38	38.77	39.27	38.88
14						38.77	38.94	38.44	38	38.72
15						38.22	39.16	38.61	39.33	38.77
16						38.61	39.05	38.61	38.61	38.72
17						38.55	39.11	38.72	38.22	38.77
18						38.72	39.38	39.11	39.11	39.27
19						39.11	39.44	38.77	38.77	39
21						38.88	39.11	38.88	39	38.77

Supplementary table S3: Classical swine fever virus (CSFV)-associated viremia in blood following CSFV-challenge in pigs. $TCID_{50}$ - 50% tissue culture infectious dose.

Group	#	CSFV-viremia in blood - Log_{10} $TCID_{50}$/ml (days post-challenge)				
		Day 0	Day 4	Day 7	Day 14	Day 21
**CSFV control**	83	<1.8	3.05	5.8	Euthanized	
	85	<1.8	3.55	5.8		
	86	<1.8	3.8	6.05		
	89	<1.8	2.8	6.05		
	90	<1.8	3.8	6.55		
	**Average**	**<1.8**	**3.4**	**6.05**		
**PRVtmv+**	84	<1.8	3.33	2.55	<1.8	<1.8
	87	<1.8	2.05	2.05	<1.8	<1.8
	88	<1.8	2.55	2.55	<1.8	<1.8
	91	<1.8	2.3	2.05	<1.8	<1.8
	92	<1.8	2.05	<1.8	<1.8	<1.8
	**Average**	**<1.8**	**2.45**	**1.84**	**<1.8**	**<1.8**

Supplementary table S4: Leukocytes count in pigs following virulent classical swine fever virus challenge in pigs.

Group	#	Leukocytes count (per µl) (days post challenge)				
		Day 0	Day 4	Day 7	Day 14	Day 21
CSFV control	83	16040	7540	5040		
	85	13240	2760	6020		
	86	12260	7300	16200		
	89	18860	5200	20480		
	90	13100	11840	10980		
PRVtmv+	84	19740	15020	20660	17240	11260
	87	25400	17060	25760	3940	10580
	88	32540	12320	27480	41680	31460
	91	17120	19900	18320	14160	11840
	92	16000	7240	9560	17060	9380

Normal leukocytes range in pigs - 11 – 22 × 10^3/µl

Supplementary table S5: Lymphocytes count in pigs following virulent classical swine fever virus challenge in pigs.

Group	#	Lymphocytes count (per ml) (days post challenge)				
		Day 0	Day 4	Day 7	Day 14	Day 21
CSFV control	83	8550	3950	1920		
	85	9560	1140	1200		
	86	6680	1940	1710		
	89	10260	2000	2050		
	90	6620	4110	1370		
PRVtmv+	84	12660	6410	10890	8590	5730
	87	12720	9990	11790	2560	5990
	88	11780	4480	6260	10360	8730
	91	11250	13360	9500	8630	7370
	92	10160	3140	3630	10770	6300

Normal lymphocytes range in pigs - $4.6 – 12 \times 10^3/\mu l$

Supplementary table S6: Platelets count in pigs following virulent classical swine fever virus challenge in pigs.

Group	#	Platelets count (per µl) (days post challenge)				
		Day 0	Day 4	Day 7	Day 14	Day 21
CSFV control	83	358000	224000	177000		
	85	439000	29000	222000		
	86	644000	164000	81000		
	89	479000	194000	143000		
	90	375000	197000	217000		
PRVtmv+	84	456000	112000	375000	93000	159000
	87	422000	412000	281000	33000	231000
	88	520000	97000	187000	418000	287000
	91	372000	503000	415000	427000	349000
	92	585000	353000	434000	550000	377000

Normal platelets range in pigs - 200 to 500 × 10^3/µl
